# Discordant Responses Between Primary Head and Neck Tumors and Nodal Metastases Treated With Neoadjuvant Nivolumab: Correlation of Radiographic and Pathologic Treatment Effect

**DOI:** 10.3389/fonc.2020.566315

**Published:** 2020-12-02

**Authors:** Dante J. Merlino, Jennifer M. Johnson, Madalina Tuluc, Stacey Gargano, Robert Stapp, Larry Harshyne, Benjamin E. Leiby, Adam Flanders, Ralph Zinner, Rita Axelrod, Joseph Curry, David M. Cognetti, Kyle Mannion, Young J. Kim, Ulrich Rodeck, Athanassios Argiris, Adam J. Luginbuhl

**Affiliations:** ^1^ Department of Otolaryngology – Head and Neck Surgery, Thomas Jefferson University, Philadelphia, PA, United States; ^2^ Department of Medical Oncology, Thomas Jefferson University, Philadelphia, PA, United States; ^3^ Department of Pathology, Thomas Jefferson University, Philadelphia, PA, United States; ^4^ Department of Pharmacology and Experimental Therapeutics, Thomas Jefferson University, Philadelphia, PA, United States; ^5^ Department of Radiology, Thomas Jefferson University, Philadelphia, PA, United States; ^6^ Department of Otolaryngology- Head and Neck Surgery, Vanderbilt University, Nashville, TN, United States; ^7^ Department of Dermatology and Cutaneous Biology, Thomas Jefferson University, Philadelphia, PA, United States

**Keywords:** squamous cell carcinoma of head and neck, nivolumab, immunotherapy, lymph nodes, computed tomography imaging

## Abstract

PD-1 blockade represents a promising treatment in patients with head and neck squamous cell carcinoma (HNSCC). We analyzed results of a neoadjuvant randomized window-of-opportunity trial of nivolumab plus/minus tadalafil to investigate whether immunotherapy-mediated treatment effects vary by site of involvement (primary tumor, lymph nodes) and determine how radiographic tumor shrinkage correlates with pathologic treatment effect.

**Patients and Methods:**

Forty-four patients enrolled in trial NCT03238365 were treated with nivolumab 240 mg intravenously on days 1 and 15 with or without oral tadalafil, as determined by random assignment, followed by surgery on day 31. Radiographic volumetric response (RVR) was defined as percent change in tumor volume from pretreatment to posttreatment CT scan. Responders were defined as those with a 10% reduction in the volume of the primary tumor or lymph nodes (LN). Pathologic treatment effect (PTE) was defined as the area showing fibrosis or lymphohistiocytic inflammation divided by total tumor area.

**Results:**

Sixteen of 32 patients (50%) with pathologic evidence of LN involvement exhibited discordant PTE between primary sites and LN. In four patients with widely discordant adjacent LN, increased PTE was associated with increased infiltration of tumor CD8^+^ T cells and CD163^+^ macrophages, whereas stromal regulatory T cells were associated with low nodal PTE. RVR correlated with PTE at both primary tumor (slope = 0.55, *p* < 0.001) and in LN (slope = 0.62, *p* < 0.05). 89% (16/18) of radiographic non-responders with T1–T3 primary sites had no (n = 7) or minimal PTE (n = 9), whereas 15/17 (88%) of radiographic responders had moderate (n = 12) or complete (n = 3) PTE.

**Conclusion:**

Nivolumab often induces discordant treatment effects between primary tumor sites and metastatic lymph nodes within subjects. This treatment discordance was also demonstrated in adjacent lymph nodes, which may correlate with local immune cell makeup. Finally, although these data were generated by a relatively small population size, our data support the use of early radiographic response to assess immunotherapy treatment effect in HNSCC.

## Introduction

Immunotherapy with the use of checkpoint inhibitors targeting PD-1 has demonstrated survival benefit in the first-line and second-line treatment of recurrent/metastatic head and neck squamous cell carcinoma (HNSCC) (1–3). Unfortunately, however, to date these advances have failed to generate sustained responses in the majority of patients.

The rapidly-growing interest in PD-1/PD-L1 inhibition for HNSCC and the use of neoadjuvant therapy underscores the unmet need for validated methods to accurately assess treatment response. The gold standard method to evaluate treatment efficacy remains surgical resection and pathologic examination of tumor. An ideal surrogate measure would correlate with pathologic treatment effect, be minimally invasive, permit rapid and inexpensive tumor evaluation, and be compatible with longitudinal tracking of tumor response. The Response Evaluation Criteria in Solid Tumors (RECIST) have been developed to provide a uniform methodology to assess tumor response to therapy relying primarily on imaging modalities (4).

For immunotherapeutic agents, including PD-1/PD-L1 inhibitors, a standardized set of consensus guidelines called “iRECIST” has been specifically designed (5). As the iRECIST consensus guidelines are not specific to HNSCC, studies relating radiographic imaging to pathology are needed. Indeed, in 2019 the Society for Immunotherapy of Cancer failed to reach consensus on the most appropriate radiographic method for quantifying HNSCC treatment response (6). Moreover, the 2019 iRECIST guidelines recommend “response assessment every 6–12 weeks”, which falls outside the timeframe of many window-of-opportunity trials (WOT), including the one investigated here (5). In this study, we follow individual lesions over a short course of immunotherapy by CT, and then compare CT results to the treatment effect demonstrated by pathological examination of resected tumor tissues. By comparing treatment effects *within* a patient, this format attempts to limit the challenges to treatment response assessment created by tumor heterogeneity and patient variability. Indeed, this approach has been validated for HPV^+^ HNSCC treated with traditional platinum-based chemotherapies prior to definitive resection (7).

We conducted an analysis of our results from a prospective preoperative window-of-opportunity trial (WOT) of nivolumab with or without tadalafil to examine whether response differs between different tumor sites and whether radiographic response correlates with pathology findings. The primary endpoint and main results of the clinical study, including biomarker analysis, will be reported separately.

## Methods

### Patients

Following approval by the Institutional Review Boards of participating institutions, 44 patients with newly diagnosed, resectable HNSCC were enrolled in a WOT (NCT03238365) and had evaluable imaging and pathology for analysis. Within 28 days of enrollment patients had undergone baseline pretreatment and CT scan, as well as a biopsy of their primary tumor. Following 4 weeks of treatment, patients underwent a second CT, followed by same-day surgical resection of the primary tumor and lymph node dissection. Patient demographics and tumor details are listed in **Supplementary Table 1 (S1)**. All patient data were pooled, regardless of treatment status with tadalafil.

### Treatment

All patients enrolled in the study received nivolumab 240 mg intravenously every 2 weeks for 2 doses (Opdivo, Bristol-Myers Squibb, NY, USA). To assess the effects of PDE5 inhibition on nivolomab efficacy, on day 0 patients were randomly assigned 1:1 to receive either nivolumab alone or nivolumab plus the PDE5 inhibitor tadalafil (Cialis, Eli Lilly, IN, USA), 10 mg orally once per day.

### Pathologic Evaluation

**Figure 1:**
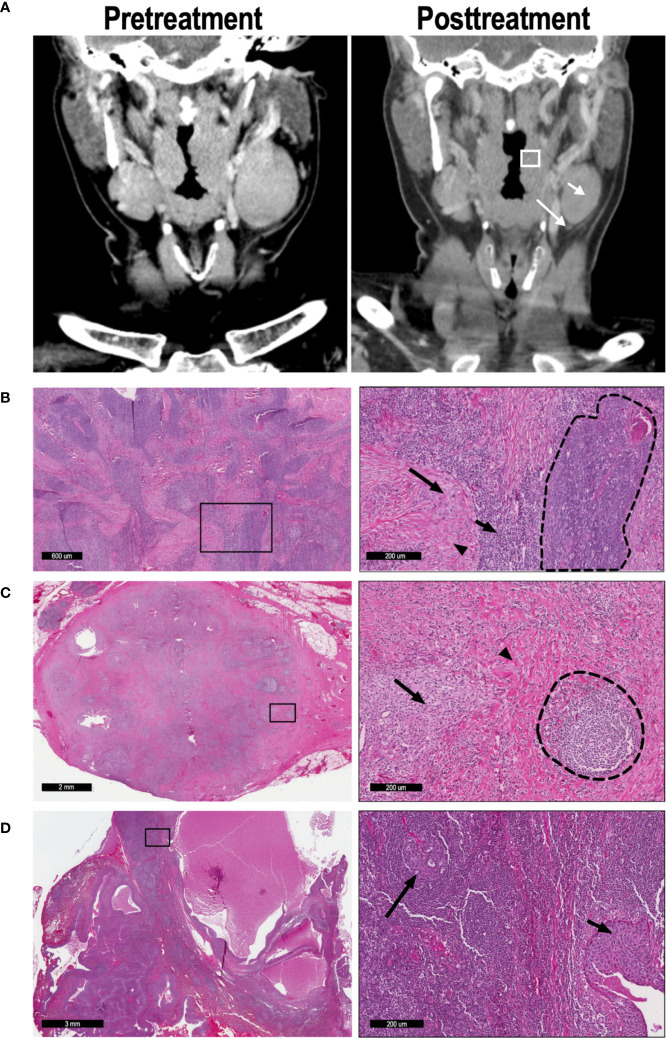
57 y/o M with P16+, cT2N2 oropharyngeal SCC. **(A)** CT imaging was performed on day 0 (pretreatment) and day 31 (posttreatment) to determine radiographic treatment effect; representative coronal imaging demonstrates the primary tumor (box), lymph node A (short arrow), and lymph node B (long arrow). **(B)** Low magnification (left) and high magnification (right) imaging demonstrates a tumor with partial (52%) pathologic treatment effect. In the high magnification image, a nest of viable tumor cells (dashed line) is bordered by lymphocytic infiltration (short arrow), macrophages (long arrow), and fibrosis (arrowhead). **(C)** Lymph node A demonstrated 100% pathologic TE, with no tumor cells evident in the node. High-powered magnification (right) reveals a poorly-formed granuloma (arrow) and extensive fibrosis (arrowhead), leading to distortion of the normal LN architecture. A residual germinal center is also present (dashed lines). **(D)** Lymph node B exhibited 0% pathologic TE, as seen in the high-magnification image (right). The normal lymph node architecture is preserved (long arrow), and a nest of malignant cells confirms tumor invasion (short arrow).

Following surgical resection of primary site and lymph nodes on day 31, specimens were processed and analyzed by two head and neck pathologists. The primary specimen and all abnormal lymph nodes were sectioned in their entirety, and all slides containing tumor were included in the analysis. Pathologic treatment effect (PTE) was defined as the area of tissue exhibiting predefined histologic criteria of tumor response as a percentage of total tumor area (S2) (8). Tumors with PTE of ≥20% were defined as “moderate” responders, while those with PTE <20% were defined as “minimal” responders. LN with 0 or 100% PTE were defined as pathologic “non-responders” or “complete responders”, respectively. Histologic analysis of primary tumor (**Figures 1A, B**) and lymph nodes (**Figures 1B, C**) reveal the criteria used to define the pathologic treatment effect. For example, **Figure 1C** demonstrates a LN with a 100% PTE, with no evidence of tumor and robust markers of inflammation, macrophage reaction, multinucleated giant cells, and granulomas. In contrast, **Figure 1D** shows an adjacent LN from the same patient with 0% PTE, with visible nests of tumor still present and no evidence of fibrosis or chronic inflammation.

### Immunohistochemistry

To investigate potential differences in the tumor microenvironment (TME) between adjacent lymph nodes with disparate treatment effects, surgical specimens were stained using antibodies to PD-L1 (clone E1L3N 1:50, Cell Signaling, MA, USA), CD8 (clone SP57 prediluted, Roche, Switzerland) CD163 (clone MRQ-26 prediluted, Roche) or FoxP3 (clone SP97 1:100, Spring Bioscience, CA, USA) to determine the number of positive cells per high-powered field. For each tumor assessed, image analysis was performed at three different regions of interest (ROI) (tumor-stromal interface, tumor, and stroma). For each ROI, three separate areas representing the highest concentrations of target cell population were analyzed per slide. Quantitative analysis of CD8, CD163, and FoxP3 was performed using Visiopharm (Visiopharm, Denmark), utilizing a linear type Bayesian classification to determine cell positivity. PD-L1 expression was performed semi-quantitatively, with cell counts determined by pathologist interpretation (<1, 1–20, and >20% TPS score). Importantly, patients receiving tadalafil were excluded from all TME analysis, in order to avoid potential confounding effects of tadalafil on TME.

### Radiographic Evaluation

Pretreatment CT scans (CT_i_) and posttreatment CT scans (day of surgery) (CT_f_) were compared to determine radiographic volumetric response (RVR). Both the primary tumor site as well as any lymph nodes identified as abnormal on the pretreatment scan were categorized as tumors and were measured using a modified radiographic evaluation to account for the short course of the neoadjuvant treatment. For each tumor, diameter was measured in three dimensions to calculate tumor volume (V), using the equation $V=\frac{4}{3}\pi (x\;axis\;*\;y\;axis\;*\;z\;axis).$ RVR of both primary site (RVR_PS_) and lymph node(s) (RVR_LN_) was calculated as the percent change between pretreatment volume (V_i_) and posttreatment volume (V_f_) using the equation $RVR=\frac{V_{f}-V_{i}}{V_{i}}$. Patients with *n* abnormal lymph nodes received an aggregate lymph node RVR $\left(\bar{RVR_{LN}}\right)$ calculated as a weighted average according to the equation:

$$\bar{RVR_{LN}}=\frac{\left(RVR_{1}*V_{i1})+(RVR_{2}*V_{i2})+\cdots (RVR_{n}*V_{in}\right)}{V_{i1}+V_{i2}+\cdots V_{in}}*100\%$$

Primary site tumors that decreased in volume by at least 10% (RVR_PS_ ≥ 10%) were categorized as “Decreased”, while primary site tumors that increased in size by at least 10% (RVR_PS_ ≤ -10%) were categorized as “Increased”; all other primary site tumors were categorized as “Stable”. These same cutoffs were also used to categorize each patient’s nodal response as “Decreased”, “Stable”, or “Increased”. These measures were combined to describe overall volumetric response (**Figures 4A, B**). Additionally, treatment response of individual primary tumors and lymph nodes were quantified and correlated to their pathologic findings (**Figures 4C, D**).

### Statistical Analysis

The association of RVR and pathologic TE correlation was evaluated using linear regression analysis for primary tumor data and using mixed effects linear regression for LN data to account for correlation among multiple observations from some subjects. RVR and PTE were transformed using logit transformation prior to analysis. Values of 100% were set to 99.9% and values of 0% were set to 0.1% prior to applying the logit transformation. All RVR demonstrating tumor growth were coded as RVR = 0%. The slope of the linear regression line on the logit-transformed data was the estimate of association. For plotting, the estimated curve was back-transformed to the percentage scale. Average PTE between primary site and lymph nodes were compared by unpaired, two-tailed t-test. Regression analysis was performed using SAS 9.4 and SAS/STAT 15.2 (SAS Institute, Cary, NC, USA). Graphs were designed using Graphpad Prism 7 software (GraphPad Software, CA, USA).

## Results

### Discordant TE Between Primary Site and Lymph Nodes Suggests Increased Therapeutic Response at Lymph Nodes Compared to Primary Tumor

**Figure 2:**
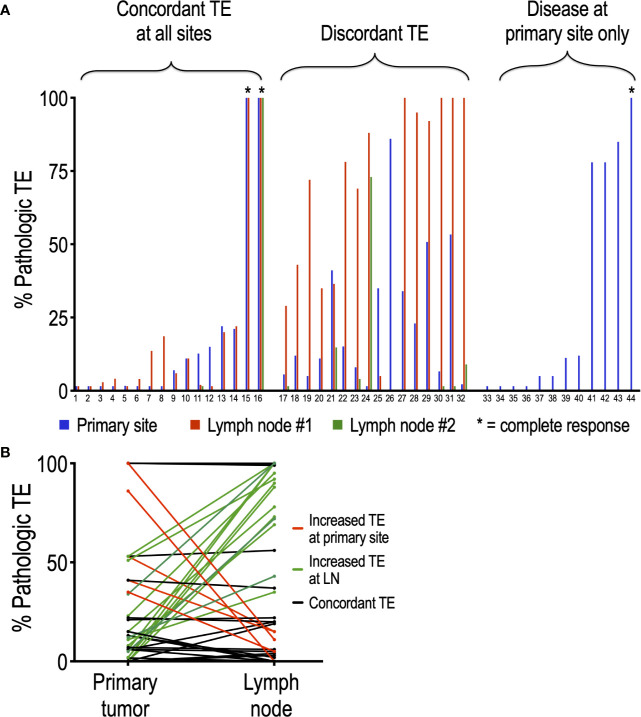
Analysis of individual patients reveals frequent discordance between pathologic TE and primary site and lymph nodes. **(A)** Of the 44 patients analyzed, 32 had lymph nodes positive in addition to primary site; 16 of the 32 (50%) patients exhibited discordant PTE. **(B)** Discordant TE is driven primarily by an increased PTE at LN relative to primary site. Of the 40 nodes analyzed, only 5 (12.5%) (red lines) had higher PTE than their primary site; conversely, 14 (35%) (green lines) nodes had higher PTE than their matched primary site. Nodes with concordant PTE at primary site are depicted in black.

Of the 44 patients evaluated in this study, 32 presented with disease in the lymph nodes in addition to the primary site. To determine whether nivolumab differentially affects tumor regression at the primary tumor site compared to lymph nodes, we compared matched pathologic TE by site within patients. Interestingly, half (16/32, 50%) of patients with regional disease exhibited discordant TE, defined by a difference in PTE of at least 20% (**Figure 2A**). Moreover, this discordance was driven by an increased TE in LN (average TE +/- SEM: 42.2 +/-6.8 %) compared to primary site (average TE +/- SEM: 26.3 +/- 5.1%) (p = 0.05). In support of this, 14 of the 40 lymph nodes analyzed (35%) had a higher TE than their matched primary site, while only 5 of 40 (12.5%) nodes had lower TE than their matched primary site (**Figure 2B**). Chi square analysis demonstrated that tadalafil did not significantly alter the rate of discordant TE compared to patients receiving placebo (44% discordance in tadalafil-treated group versus 60% discordance in placebo group, p = 0.373).

### Comparison of Tumor Microenvironment Between Matched Lymph Nodes With Widely Discordant Pathologic TE

**Figure 3:**
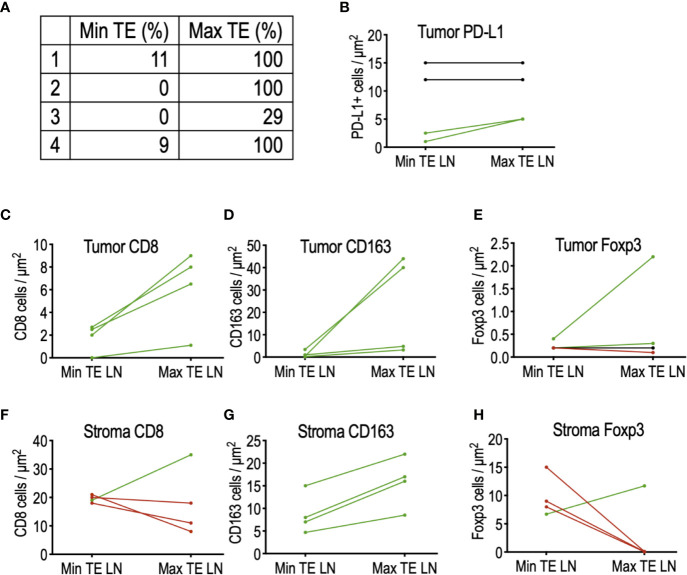
Comparison of the tumor microenvironment in matched lymph nodes of four patients with highly discordant PTE. **(A)** List of matched lymph node PTE. **(B)** PD-L1 expression did not appear to influence PTE between discordant LNs. **(C)** Histologic analysis of tumor-infiltrating immune cells demonstrated a trend toward increased CD8 T cells and **(D)** increased CD163^+^ macrophages in LN with maximal PTE; **(E)** no detectable trend was detected in tumor-infiltrating FoxP3^+^ Tregs. **(F)** No detectable relationship was found between stromal CD8 T cells and PTE, but **(G)** stromal CD163^+^ macrophages were increased in LN with maximal TE. **(H)** 75% of the max TE LN contained no stromal FoxP3^+^ Tregs, in sharp contrast to their matched min TE LN.

Interestingly, analysis of patients not receiving tadalafil revealed a small cohort of four patients exhibiting widely disparate PTE between adjacent lymph nodes, including one patient with complete resolution of tumor in one node and absence of any TE in an adjacent node (**Figure 3A**). To determine whether this was due to differences in the immune microenvironment, CD8^+^ cytotoxic T cells, FoxP3^+^ Treg cells, and CD163^+^ macrophages were quantified in the tumor and surrounding stroma of the LNs of interest (**Figures 3B–H**). Although this cohort is too small to draw conclusions, comparison of immune infiltrates in these matched nodes revealed a trend toward increased CD8^+^ T cells (**Figure 3C**) and CD163^+^ macrophages (**Figure 3D**) in tumors of nodes with increased TE, while increased stromal Tregs (FoxP3^+^) were associated with nodes demonstrating reduced TE (**Figure 3H**). Interestingly, there did not appear to be any relationship between PD-L1 staining intensity and TE (**Figure 3B**).

### Radiographic Volumetric Response Correlates With Pathologic TE

**Figure 4:**
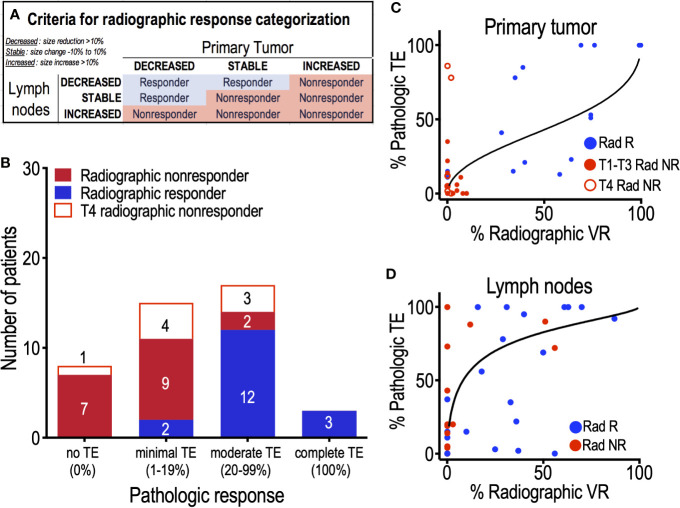
Correlation of radiographic volumetric response and pathologic treatment effect. **(A)** Assignment of patients to radiographic volumetric response groups, based on the percent change in volume of both the primary tumor and the combined volume of all abnormal lymph nodes. **(B)** All 8 patients with T4 primary tumors (red outlines) were characterized as radiographic non-responders. 18 patients with T1–T3 primary sites were designated radiographic non-responders (solid red); 89% (16/18) exhibited no pathologic treatment effect (*n* = 7) or minimal PTE (*n* = 9). Of the 17 patients designated radiographic responders (blue), 15 (88%) demonstrated moderate (*n* = 12) or complete (*n* = 3) treatment effect. **(C)** Quantitative analysis of primary sites demonstrates a strong positive correlation between RVR and pathologic TE (slope = 0.55, *p* < 0.001) of individual tumors, despite the failure of T4 primary tumors (red outlined circles) to demonstrate any RVR. **(D)** Analysis of abnormal nodes demonstrates a significant correlation (slope = 0.65, *p* < 0.05). For **(D)**, each data point represents a single node.

We next sought to determine whether changes in tumor size quantified by CT accurately reflected true treatment efficacy in eliminating tumor cells. To do this, we first categorized each patient as a radiographic responder or non-responder based on the change in size of both the primary tumor as well as any abnormal lymph nodes (**Figure 4A**). We then compared these results to the patient’s PTE, the gold standard of treatment effect determination. Interestingly, all eight patients with T4 primary tumors were categorized as radiographic non-responders, despite the fact that 38% (3/8) of T4 tumors demonstrated moderate PTE. Conversely, only 11% (2/18) of “radiographic non-responder” patients with T1-T3 primary tumors exhibited moderate pathologic treatment effect. Among T1-T3 “radiographic responder” patients, 88% (15/17) exhibited moderate (> 20% PTE) or complete PTE. This suggests that radiographic quantification of treatment response may not be effective in patients with T4 primary tumors.

Quantitative analysis of individual tumor sites demonstrated a significant correlation between RVR and pathologic treatment effect. This correlation between RVR and PTE was significant both at the primary tumor site (**Figure 4C**) (slope = 0.55; 95% CI 0.25, 0.85; *p* < 0.001) as well as at metastatic lymph nodes (**Figure 4D**) (slope = 0.65; 95% CI 0.22, 1.09; *p* < 0.05).

One patient had newly enlarged lymph nodes without necrosis or abnormal shape on the posttreatment CT and the final pathology revealed these lymph nodes to be benign.

## Discussion

Our study investigated HNSCC patients treated with a brief course of neoadjuvant nivolumab prior to surgical resection and evaluated treatment effect by CT imaging as well as by pathology. Because most patients included in this study had multiple sites of malignancy [primary and lymph node(s)], we had the opportunity to compare treatment effect of nivolumab within individual patients. This analysis revealed that patients with multiple sites of disease frequently exhibit discordant responses, demonstrating that even within a patient the tissue subtype and tumor microenvironment (TME) plays a substantial role in nivolumab efficacy. Moreover, our finding that pathologic lymph nodes frequently displayed more favorable treatment effect than the primary site suggests that immune cell concentration and/or cytokine makeup could influence the efficacy of nivolumab; future studies are needed to determine whether this is the case. Indeed, a 2017 study of the TME by Jimenez-Sanchez et al. revealed that the TME may influence tumor progression between metastases within a patient (9). This is hinted by the comparison of nodes with discordant treatment effects, which demonstrated an increased number of tumor-infiltrating CD8 T cells and CD163^+^ macrophages and decreased stromal FoxP3^+^ Tregs in nodes with 100% TE. This hypothesis-generating study suggests that further interrogation and comparison of the TME between responding and nonresponding tumor sites within a patient may provide valuable insight into the mechanisms of tumor resistance to checkpoint inhibition. Further investigation of the TME is necessary to understand the mechanism underlying these findings and determine whether they play a role in resistance to immunotherapies such as nivolumab.

We also demonstrate that in 4 weeks of treatment, RVR for individual tumors correlates with pathologic TE, both at primary sites (slope = 0.55) as well as at metastatic lymph nodes (slope = 0.65). Moreover, we describe a radiographic algorithm that detected minimal or no pathologic response to nivolumab (defined by pathologic TE <20%) with a negative predictive value (NPV) of 81% (21/26); excluding patients with T4 primary tumors increased this NPV to 89% (16/18). Conversely, the algorithm detected moderate or complete pathologic treatment response (pathologic TE ≥20%) with a positive predictive value 75% (15/20) or 88% (15/17) if T4 tumors are excluded. One possible explanation for these findings is that the burden of disease in T4 tumors may slow immune cell infiltration and macrophage debris clearance, delaying radiographic evidence of tumor involution to beyond the window of time investigated. Additionally, pretreatment CT scans were performed up to 28 days prior to first nivolumab dose. While this has could result in underreporting of RVR across all tumors, additional tumor growth during this time may have been more prominent in larger, more aggressive T4 tumors. While this algorithm was generated retrospectively and therefore requires validation with additional patient populations to confirm its utility, it provides a valuable starting point by which future HNSCC tumors can be monitored over brief window trials.

By demonstrating the discordant treatment effects between primary sites and adjacent nodes, this study achieves several ends. First, it supports the findings of other studies demonstrating similar discordance in other solid malignancies, such as lung cancer (10, 11). Importantly, however, unlike the aforementioned studies, this research utilizes the gold standard of tumor progression, histopathology, to compare true treatment effect to that which is demonstrated by CT imaging. This has several important consequences. First, it highlights the potential confounder of pseudoprogression, by confirming that tumor growth on CT does not necessarily equate to expansion of malignant cells. Second, this study demonstrates that this pseudoprogression may be particularly prominent in large, bulky disease with T4 primary lesions. This is of particular importance in head and neck cancer window trials, since many window trials do provide a long enough window of time to follow tumors until pseudoprogression can be confirmed by CT imaging. Finally, it confirms discordance at adjacent sites by histopathology shortly after administration of checkpoint inhibition, which is not possible by CT imaging alone, due to the need for repeat imaging to rule out pseudoprogression.

Landmark studies by Ferris et al. in 2016 (3) and Cohen et al. in 2019 (2) showed that treatment with PD-1 inhibitors (nivolumab or pembrolizumab) increased overall survival in patients with platinum-refractory recurrent or metastatic HNSCC. More recently, the results of KEYONOTE-048 established pembrolizumab +/- chemotherapy as the standard of care in the first-line treatment of recurrent or metastatic HNSCC. However, only a small fraction of patients with HNSCC derives benefit from immunotherapy. (2) With a multitude of new studies expected in the near future, this work underscores the importance of understanding response patterns in HNSCC and that developing and validating early non-invasive assessment of response may inform treatment decision-making as well as assist in the evaluation of immunotherapy efficacy.

There are several limitations to this study. First, the algorithm utilized to categorize patients as radiographic responders/non-responders was generated after patient data were collected. The efficacy of this algorithm to detect patients with treatment failure will need to be validated by applying the algorithm in a prospective trial. Second, the sample size of 44 individuals analyzed is relatively low, limiting the ability to extrapolate these findings across patient populations. The small cohort of four patients with discordant LN TE prevents quantitative assessment of the data generated, limiting the findings to qualitative trends. Finally, although tadalafil was not found to significantly alter the rate of discordant TE at different tumor sites, this does not definitively eliminate the possibility of treatment-mediated confounding effects. Despite these limitations, our observations add important context to the growing body of literature describing HNSCC treated with immunotherapy, and lays the foundation for future studies that can build off of these findings. We plan to further correlate both CT as well pathologic TE findings with immune biomarkers from blood and tissue.

Because of its design as a window-of-opportunity trial, the duration of pharmacologic treatment (4 weeks) is significantly less than the treatment settings in which RECIST are commonly applied. (5) However, window trials represent an important tool in evaluating the efficacy of novel HNSCC treatment regimens, allowing patients access to novel therapies without delaying definitive surgical therapy. Indeed, numerous window trials for HNSCC exist in the literature, including neoadjuvant treatment with EGFR inhibition (12–15) and/or immunotherapy (16, 17). A recent study by Sadeghi et al. (7) demonstrated a correlation between pathologic and radiographic responses in HPV+ oropharyngeal cancers in the context of a window-of-opportunity trial with neoadjuvant cisplatin/docetaxel. Our study advances this work by providing additional data on a broader range of HNSCC subtypes, and to our knowledge is the first such analysis of patients treated with an immune checkpoint inhibitor.

In conclusion, we demonstrated discordant responses between primary tumor and regional metastatic lymph nodes after brief neoadjuvant preoperative nivolumab therapy in HNSCC. We suggest that this may correlate with variations in local immune response. Moreover, our study demonstrated that neoadjuvant immunotherapy can induce rapid, quantifiable changes in tumor volumes assessed by CT scan. We applied novel radiographic volumetric response criteria and showed that radiographic response correlates with pathologic treatment effect. We propose that future neoadjuvant window of opportunity trials with immune checkpoint inhibitors incorporate and further validate these radiographic response criteria.

## Data Availability Statement

The raw data supporting the conclusions of this article will be made available by the authors, without undue reservation.

## Ethics Statement

The studies involving human participants were reviewed and approved by the Institutional Review Board at Thomas Jefferson University Hospital and Institutional Review Board at Vanderbilt University Medical Center. The patients/participants provided their written informed consent to participate in this study.

## Author Contributions

DM took part in the formal analysis, visualization, writing the original draft, and writing reviewing, and editing the manuscript. JJ was involved in the conceptualization, investigation, formal analysis, and writing, reviewing, and editing the manuscript. MT took part in the formal analysis, data curation, and writing, reviewing, and editing the manuscript. SG was involved in the formal analysis, data curation, and writing, reviewing, and editing the manuscript. RS was in charge of the formal analysis, data curation, and writing, reviewing, and editing the manuscript. LH took part in the formal analysis, and writing, reviewing, and editing the manuscript. BL was in charge of the formal analysis, software, validation, and writing, reviewing, and editing the manuscript. AF was in charge of the data curation, formal analysis, software, and writing, reviewing, and editing the manuscript. RZ took part in the formal analysis, and writing, reviewing, and editing the manuscript. RA took part in the formal analysis, and writing, reviewing, and editing the manuscript. JC was in charge of the resources, data curation, and writing, reviewing, and editing the manuscript. DC was in charge of the resources, data curation, and writing, reviewing, and editing the manuscript. KM was in charge of the resources, data curation, and writing, reviewing, and editing the manuscript. YK was in charge of the resources, data curation, writing, reviewing, and editing the manuscript, funding acquisition. UR was involved in the conceptualization, methodology, writing, reviewing, and editing the manuscript, funding acquisition. AA took part in the investigation and writing, reviewing, and editing the manuscript. AL was in charge of the project administration, supervision, methodology, conceptualization, resources, formal analysis, and writing, reviewing, and editing the manuscript. All authors contributed to the article and approved the submitted version.

## Funding

This work was supported by the National Institutes of Health (R01DE027749, R01CA178613 to YK), the Department of Defense (W81XWH-16-1-0679 to UR) and CDMRP Breakthrough Award to YK, Bristol Myers Squibb, and the Thomas Jefferson Deans Grant (to the Jefferson Squamous Cell Working Group).

## Conflict of Interest

UR serves on Scientific Advisory Board of Akrevia/Xilio. YK serves on the Scientific Advisory Board for the following companies: Aduro, Sanofi, Takeda, Mersanna, and Dracen. Additionally, Bristol Myers Squibb contributed funding to this study, but did not participate in data collection, analysis, or patient recruitment.

The remaining authors declare that the research was conducted in the absence of any commercial or financial relationships that could be construed as a potential conflict of interest.
