# Supplementary material for: Discordant Responses Between Primary Head and Neck Tumors and Nodal Metastases Treated With Neoadjuvant Nivolumab: Correlation of Radiographic and Pathologic Treatment Effect
Source: Front Oncol. 2020 Dec 2;10:566315. doi: 10.3389/fonc.2020.566315 (PMC7738605; doi:10.3389/fonc.2020.566315)
Supplement: Supplementary file 1 [file Table_1.docx]

| Supplementary table 1. Patient characteristics | | | | | | |
| --- | --- | --- | --- | --- | --- | --- |
|  | All Data  (*n* = 44) | Concordant  PTE  (*n* = 16) | Discordant  PTE  (*n*=17) | | Disease at primary site only  (*n* = 11) | |
| Age (average years) | 63 | 64 | 63 | | 62 | |
| Tadalafil: Placebo | 25:19 | 10:6 | 8:9 | | 7:4 | |
| Male: Female | 41:3 | 15:1 | 16:1 | | 10:1 | |
| Primary Site |  | | | | | |
| Larynx/Hypopharynx | 6 | 0 | | 1 | | 5 |
| Oral/Nasal Cavity | 14 | 4 | | 6 | | 4 |
| Oropharynx | 24 | 12 | | 10 | | 2 |
| T Stage (clinical) |  | | | | | |
| T1 | 14 | 3 | | 7 | | 4 |
| T2 | 18 | 8 | | 7 | | 3 |
| T3 | 4 | 3 | | 0 | | 1 |
| T4a | 8 | 2 | | 3 | | 3 |
| N Stage |  | | | | | |
| N0 | 10 | 1 | | 1 | | 8 |
| N1 | 20 | 9 | | 10 | | 1 |
| N2a | 5 | 2 | | 3 | | 0 |
| N2b | 5 | 2 | | 2 | | 1 |
| N3 | 2 | 1 | | 1 | | 0 |
| Nx | 2 | 1 | | 0 | | 1 |
| Pathologic Markers |  | | | | | |
| P16 Positive | 22/44 (50%) | 12/16 (75%) | | 8/17 (47%) | | 2/11 (18%) |
