# Supplementary material for: Discordant Responses Between Primary Head and Neck Tumors and Nodal Metastases Treated With Neoadjuvant Nivolumab: Correlation of Radiographic and Pathologic Treatment Effect
Source: Front Oncol. 2020 Dec 2;10:566315. doi: 10.3389/fonc.2020.566315 (PMC7738605; doi:10.3389/fonc.2020.566315)
Supplement: Supplementary file 2 [file Table_2.docx]

| Supplementary table 2. Histologic criteria for pathologic treatment effect |
| --- |
| Macrophage reaction |
| Multinucleated giant cells and granulomas |
| Fibrosis and chronic inflammation adjacent to residual tumor nests |
| In cases of complete response: distortion of normal tissue architecture |
